# Finite element analysis-based optimization of longitudinal bending stiffness and rearfoot stability in carbon-plated running shoes

**DOI:** 10.3389/fbioe.2026.1797727

**Published:** 2026-03-30

**Authors:** Sewon Kim, Youngbin Lim, Siwoo Jung, Luca Quagliato, Olamide Robiat Hassan, Jeicheong Ryu, Taeyong Lee

**Affiliations:** 1 Division of Mechanical and Biomedical Engineering, Ewha Womans University, Seoul, Republic of Korea; 2 Division of Mechanical and Biomedical Engineering, Graduate Program in System Health Science and Engineering, Ewha Womans University, Seoul, Republic of Korea; 3 SIMULIA, Dassault Systèmes Korea, Seoul, Republic of Korea; 4 Human Performance Lab. Co., Ltd., Seoul, Republic of Korea

**Keywords:** ankle sprain, carbon-plated running shoes, finite element analysis (FEA), longitudinal bending stiffness, rearfoot stability, running shoe, running injury

## Abstract

**Introduction:**

The carbon-plated midsole in running shoes plays a pivotal role in enhancing the runner’s performance by storing and releasing energy. A key factor in running shoes is the Longitudinal Bending Stiffness (LBS), where higher LBS usually improves energy efficiency by enhancing energy return during a running cycle. However, a critical trade-off exists: excessive LBS can diminish performance and may increase the risk of injury.

**Methods:**

To this end, this study aims to model and optimize the balance between energy efficiency and stability through finite element analysis (FEA). Specifically, the LBS was systematically adjusted by varying midsole foam materials and carbon plate thicknesses. A total of two FEA models were employed: a three-point bending simulation accessing LBS, and a lateral loading model accessing rearfoot stability. Boundary conditions for both models were defined through preliminary simulations. A parametric analysis was conducted by varying the midsole foam material and carbon plate thickness to identify optimal configurations.

**Results:**

Preliminary results indicate that EVA midsoles exhibited the greatest LBS and stability, followed by PEBA, both outperforming TPU. Furthermore, thicker carbon plates showed a higher value of LBS but had little effect on stability.

**Discussion:**

This research provides a novel standard of LBS testing and a novel FEA modeling framework for designing carbon-plated running shoes that enhance performance while reducing injury risks.

## 1 Introduction

Although running is often perceived as a simple sport activity, it involves a complex interaction between the runner, the footwear, and the terrain. While running, the lower limbs, particularly the foot, experience repetitive and continuous impact loads. These loads can lead to injuries, underscoring the importance of the shock absorption ability of running shoes (Krabak, Snitily, and Milani, 2016; Scheer et al., 2022; Assumpcao Cde et al., 2013; Millet and Lepers, 2004). In this regard, a critical component of running shoes is the midsole, which works as a medium between the runner and the ground (Malisoux et al., 2020; Teoh et al., 2013; Autodesk, 2025). The midsole foam acts as a shock absorber and a spring, dissipating impact energy and storing it for propulsion during the gait cycle (Sun et al., 2020). During running, the midsole undergoes compression and bending and converts the received energy into other forms. Simultaneously, it stores energy during the heel strike phase and releases it upon unloading, thereby enhancing energy efficiency and reducing the metabolic cost of locomotion.

In recent decades, advances in material science, manufacturing, and biomechanics, have significantly improved running shoe design, particularly in terms of weight reduction (Franz, Wierzbinski, and Kram, 2012; Frederick, 1984; Hoogkamer et al., 2016), geometry optimization (Madden et al., 2016; Roy and Stefanyshyn, 2006), and material innovations (Roy and Stefanyshyn, 2006; Worobets et al., 2014; Frederick, 1986). In the field of running shoes, one of the most remarkable innovations is carbon-plated shoes, often referred to as ‘technical doping’, which brought a huge evolution. The two crucial factors of carbon-plated running shoes are highly resilient and lightweight midsole foam and a stiff carbon plate embedded in the midsole. These technical developments have improved athletic performance, as evidenced by breaking world records, including Eliud Kipchoge becoming the first human to complete a full marathon in under 2 hours in 2019.

The foam materials of running shoe midsoles have undergone significant innovation over the last decades. Ethylene-vinyl acetate (EVA) has been the most widely used midsole foam material for high energy return for several decades (Aimar et al., 2024; Van Alsenoy, Ryu, and Girard, 2019). Recently, thermoplastic polyurethane (TPU) and polyether-block-amide (PEBA) have emerged as leading alternatives, reflecting current trends in midsole foam material innovation (Van Alsenoy, Ryu, and Girard, 2019; Brückner et al., 2010; Chen et al., 2022). TPU has demonstrated increased resilience, resulting in a 4% improvement in running economy when used as a midsole compared to conventional alternatives (Hoogkamer et al., 2018). However, some studies indicate that EVA provides better running economy and comfort for runners compared to TPU, suggesting that the choice of optimal midsole foam material would vary based on specific requirements and individual preferences (Van Alsenoy, Ryu, and Girard, 2019). PEBA, the latest innovation in midsole foam materials, has been validated for its high energy return rate, delivering more than double the mechanical energy and reducing metabolic cost by 4%, compared to EVA (Rodrigo-Carranza et al., 2024).

The longitudinal bending stiffness (LBS), which describes a shoe’s ability to resist bending forces, is a crucial factor in evaluating the flexibility and rigidity of running shoes. LBS can be effectively tuned by adjusting the midsole foam material (Silva et al., 2009), geometry of the midsole (Chambon et al., 2014; Chambon, Delattre, N., Berton, E., Guéguen, N., & Rao, G. 2013) and the carbon plates (Madden et al., 2016; Flores et al., 2021; Xu et al., 2025). Running shoes with low LBS can enhance energy efficiency and providing superior cushioning, they also have significant limitations (Worobets et al., 2014). Low LBS can lead to insufficient support, which can easily result in lateral instability on uneven terrain or sudden changes in direction (Madden et al., 2016; McCormick and Anderson, 2009). At the same time, excessive compressibility can increase the range of motion in the rearfoot, impairing rearfoot control and elevating the risk of injury (H. Chen, Zhang, and Biró, 2024; Kulmala et al., 2018). In addition, running shoes with high LBS showed enhanced performance and can prevent injury by limiting excessive movement of joints (Willwacher, 2017; Stefanyshyn and Wannop, 2016; Brückner et al., 2010). However, according to previous research, excessive LBS can result in increased contact time with the ground, leading to slower running speed (Song et al., 2024) and a larger amount of impact on muscles and joints (Rodrigo-Carranza et al., 2022).

This trade-off between cushioning and control emphasizes the need for appropriate selection of LBS in shoe design to balance performance and risk of running-related injuries (Bagehorn et al., 2024; Liu et al., 2022; Tenforde et al., 2023). In summary, adjusting LBS through the midsole foam material and carbon plate is essential in optimizing energy efficiency and stability (Bagehorn et al., 2024; Liu et al., 2022; Tenforde et al., 2023). In this regard, the Finite Element Analysis (FEA) method has been employed to investigate the optimal design of running shoes. For instance, J. Hale et al. evaluated shoe tread parameters using FEA (Hale et al., 2021). Similarly, Zihan Yang et al. analyzed the effects of running shoe design on plantar pressure during heel landing through FEA combined with the Taguchi optimization method (Yang et al., 2022). Additionally, Jin-Rae Cho et al. studied the landing impact of sports shoes using an FEA model (Cho et al., 2009) and Zhou et al. examined the effects of contact angles during forefoot running using FEA simulation. Similar approaches are also central in the field of prosthetic running, where carbon fiber blades play an important role. A recent study explored the effect of blade materials on performance through finite element analysis, providing valuable insights into the optimal material selection for prosthetic running blades.

However, to the best of the authors’ knowledge, no research has explicitly explored the impact of midsole design, in terms of midsole foam material and plate thickness, on the point of both energy return and stability of running shoes. This paper presents a detailed FEA modeling approach, using Nike Vaporfly 3 as a reference, to address this gap. In this study, the optimal design of carbon-plated running shoes was systematically analyzed by adjusting the midsole foam material and the thickness of the carbon plate. Using FEA, the effects of various midsole foam materials and carbon plate thickness under three-point bending and lateral loading model were evaluated. This approach can contribute to the development of running shoes that not only enhances the performance of runners but also reduces the risk of injury, offering insights into optimizing midsole designs for athletes and shoe manufacturers.

## 2 Materials and methods

### 2.1 Geometry acquisition

**FIGURE 1:**
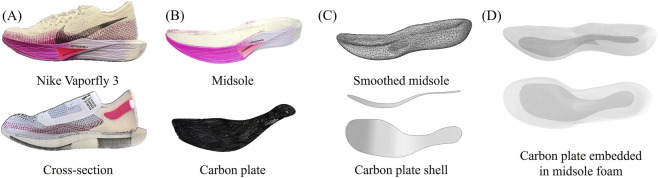
**(A)** Photo of the NVF3 and its cross-sectional view, **(B)** photos of the midsole and carbon plate, **(C)** relevant 3D scan, and **(D)** embedded midsole geometry for FEA.

In this research, the Nike Vaporfly 3 (NVF3, Nike, United States) in US M10 size (280 mm) was employed as the benchmark and reference model, as illustrated in Figure 1. The key geometrical details for the NVF3 employed in this research are reported in Supplementary Material A. The two major components of carbon-plated running shoes are the midsole foam and the carbon plate, whose 3-dimensional (3D) geometries were scanned with a Samsung Galaxy S23 (Samsung, South Korea) with Polycam software (Polycam Inc., United States). To obtain the midsole foam geometry, the upper part of the NVF3 and the insole were carefully removed from the shoe, by unpicking the stitches to avoid any deformation or damage to the midsole structure. The outsole, which is about 2 mm thick, was not removed prior to scanning. This decision was made to preserve the midsole geometry, which is soft foam and could be easily deformed or damaged during outsole removal. Given the outsole’s minimal thickness and its limited influence on the overall midsole geometry, any residual effects can be digitally post-processed. The obtained 3D geometry was subsequently smoothed by Fusion 360 (Autodesk Inc., United States) to minimize distortion created during the scanning process (Autodesk. Fusion 360 Help Documentation. San Francisco, CA: Autodesk, 2025). Afterward, the top and bottom parts of the midsole were mechanically removed, leaving only the carbon plate part, subsequently scanned and converted into a shell using the same software and technique. Both midsole and carbon plate parts were then input into the Abaqus 2025 environment, a FEA software widely used for simulating mechanical behavior of materials.

### 2.2 Material modeling

The material properties of the carbon plate, including an elastic modulus of 33,000 MPa, a Poisson’s ratio of 0.4, and a mass density of 1,100 kg/m^3^, were adopted from a previous study (Song et al., 2023). However, in Abaqus, elastic material properties are valid only for small strain levels, typically less than 0.05 (Corp., Dassault Systèmes Simulia, 2025a). For materials that undergo large deformations, such as foams, hyperelastic material modeling is generally required to capture their nonlinear responses. To address this limitation, the elastic mechanical properties of the carbon plate were converted into equivalent hyperelastic material model constants using the Neo-Hookean model, which can enable accurate under large strain conditions (Corp., Dassault Systèmes Simulia, 2025b). The corresponding strain energy potential is defined in Equation 1, where *W* is the strain energy density, *μ* is the shear modulus, *I*_1_, is the deviatoric part of first invariant of the right Cauchy-Green deformation tensor, *K* is the bulk modulus, and *J* is the determinant of the deformation gradient. Conversion to a hyperelastic material model from elastic material properties was conducted through Equation 2, where *C*_10_ is the first-order polynomial coefficient in the strain energy function, *E*_0_ is the elastic modulus of the material, *v*_0_ is Poisson’s ratio of the material and *D*_1_ is the volumetric coefficient. The relevant hyperelastic material properties of the carbon plate are summarized in Table 1.

$$W=\frac{\mu }{2}\left(I_{1}-3\right)+\frac{2}{K}{\left(J-1\right)}^{2}$$ (1)

$$C_{10}=\frac{E_{0}}{4\left(1+v_{0}\right)},\;D_{1}=\frac{6\left(1-2v_{0}\right)}{E_{0}}$$ (2)

**TABLE 1:** Elastic and equivalent hyperelastic material properties of the carbon plate.

Elastic	Elastic modulus	Poisson’s ratio	Density
	33.0 GPa	0.4	1,100 kg/m^3^
Hyperelastic (Neo-Hookean)	*C*_10_	*D*_1_	Density
	5.89 GPa	3.63 × 10^−5^ MPa^-1^	1,100 kg/m^3^

**FIGURE 2:**
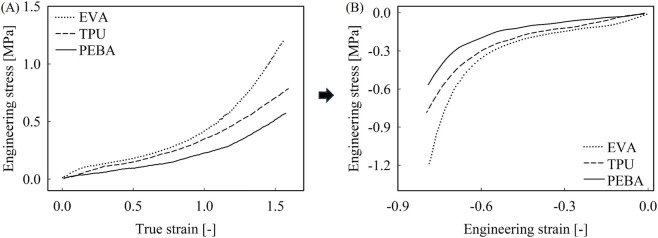
Stress-strain behavior of midsole foam materials. **(A)** Engineering stress-true strain curves from the reference. **(B)** Derived compressive engineering stress-strain curves.

For midsole foam materials, a total of three materials from various running shoe midsoles were adopted from the reference, representing different material compositions: EVA, TPU, and PEBA (Aimar et al., 2024). The compressive stress-strain behavior from the reference was initially represented using Hencky strain and engineering compressive stress. Hencky strain, also referred to as true strain or logarithmic strain, is a commonly employed method in large deformation analyses, particularly in the modeling of hyperelastic materials (Xiao and Chen, 2002). However, most hyperelastic formulation is based on the stretch ratio (*λ* = 1+*ε*_eng_) rather than true strain including Abaqus. Therefore, these stress-strain curves were converted into engineering stress-strain curves according to Equation 3, with the resulting stress-strain curves are presented in Figure 2.

$$\varepsilon =\left|\ln\left(\frac{h}{h_{0}}\right)\right|=\left|\ln\left(1+\frac{\delta }{h_{0}}\right)\right|\frac{\delta }{h_{0}}=e^{-\varepsilon }-1$$ (3)

where,

$$W=\frac{\mu^{1}}{\alpha^{1}}\left({\bar{\lambda }}_{1}^{\alpha_{1}}+{\bar{\lambda }}_{2}^{\alpha_{1}}+{\bar{\lambda }}_{3}^{\alpha_{1}}-3\right)+\frac{1}{D_{1}}\left(J^{-2\gamma_{1}}-1\right)$$ (4)

Using the Abaqus/CAE material evaluation feature, the data was fitted to a 1st order hyperfoam model, whose strain energy potential is defined in Equation 4, where *μ*_1_ is the shear-like modulus, and *α*_1_ is a dimensionless parameter that governs the nonlinear stiffening behavior of the material under deviatoric deformation. The terms ${\bar{\lambda }}_{i}$ (for *i* = 1,2,3) are the principal stretches, *J* is the determinant of the deformation gradient, indicating volumetric change. *D*_1_ and *γ*_1_ are compressibility parameters that govern the material’s volumetric response (Thomson et al., 1999). The material properties of foams were derived exclusively from compressive test data obtained from reference (Aimar et al., 2024). This is consistent with related studies on polyurethane foams, which have also relied solely on compression data for material characterization (Bezazi and Scarpa, 2007). However, to assess the robustness of the fitted hyperfoam model, the engineering stress-strain responses were evaluated under uniaxial, planar and biaxial deformation modes. This approach ensured the model’s stability across a range of loading conditions, despite being calibrated solely with compressive data. The first order hyper foam model demonstrated stability across the entire strain range, validating its suitability for characterizing the midsole foam materials under various loading conditions. This indicates that higher-order strain energy potentials were not further required. The compressive engineering stress-strain curves based on true strain and engineering strain of each material and Poisson’s ratio of each material are summarized in Table 2.

**TABLE 2:** Material properties of three different midsole foam materials.

Midsole Foam Material	Density [kg/m^3^]	Model coefficients (1st order hyperfoam)			References
		*μ*_1_ [MPa]	*α*_1_	*ν*_1_	
EVA	170	0.148	3.595	0.2	Aimar et al. (2024); Thomson et al. (1999)
TPU	240	0.0878	2.11	0.25	Aimar et al. (2024); Bezazi and Scarpa (2007)
PEBA	90	0.112	5.05	0.3	Vasileiou et al. (2023); Aimar et al. (2024)

### 2.3 Finite element simulation

This study proposes two FEA simulations: bending and lateral loading. First, the bending simulation was designed to evaluate the LBS of the running shoe midsole. A three-point bending method, a traditional approach for assessing LBS, was employed. Second, lateral loading model, newly proposed in this study, was developed to evaluate the lateral rearfoot stability of the running shoe. This simulation was designed to replicate the ankle sprain scenarios during running. The detailed information regarding each model is summarized in the following sections.

#### 2.3.1 Numerical settings

A dynamic explicit solver under quasi-static conditions was employed for both the bending and lateral loading models. The nonlinear geometry option was enabled to accurately capture the large deformations of the hyperelastic carbon plate and hyperfoam midsole foam, ensuring the accurate stress deformation under finite strain conditions. To validate the quasi-static approximation, which assumes that inertia can be ignored, the kinetic energy of the entire model was maintained below 5% of the internal energy throughout the simulation (Lee et al., 2024), with the critical time increment set to 5x10^−6^ s via mass scaling. Although both simulations were conducted for a total simulation time of 1 s, the simulation time does not correspond to a physical time due to the quasi-static assumption. The general contact in Abaqus/Explicit solver was adopted for the contact simulation, which can automatically define all possible contact pairs (Quagliato et al., 2025a). The Coulomb friction model with a friction coefficient µ = 0.6 was applied at the contact between the midsole and other parts (Fu et al., 2021). Furthermore, to prevent the midsole from vibrations, viscous pressure load in Abaqus/Explicit was employed (Lee et al., 2024) to dissipate the inertial energy to achieve static equilibrium.

The midsole foam was meshed using solid linear tetrahedron C3D4 elements, while the carbon plate was meshed with linear triangular S3R shell elements. The total number of elements was 110,954 for the midsole foam and 1,336 for the carbon plate. The carbon plate was embedded into the midsole foam at the location, which is an identical location to the actual NVF3, by using the embedded region constraint of Abaqus. The embedded element technique specifies a group of elements that lie embedded in a group of host elements whose response will be used to constrain, effectively embedding the smaller structure into the larger structure (Tabatabaei, Lomov, and Verpoest, 2014). In this approach, the embedded element is fully constrained by the host element, meaning no relative motion or friction is allowed, thus perfect bonding applies (Tabatabaei, Lomov, and Verpoest, 2014). This assumption is based on strong adhesion between carbon plate and midsole foam, which is typically achieved during manufacturing. While delamination or slip may occur due to fatigue or prolonged wear, replicating such phenomena would require a contact-based modelling approach rather than the embedded element method. This would necessitate creating an exact cavity in the host structure, defining appropriate contact properties (e.g., friction coefficient), and reprocessing the geometry whenever the plate’s position or shape changes. Given these complexities and the focus of the current work, the embedded element method which does not require additional geometry modification with a perfect bonding assumption was adopted.

#### 2.3.2 Bending model–longitudinal bending stiffness

**FIGURE 3:**
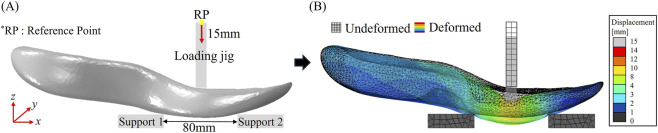
**(A)** Boundary conditions of the 3-point bending model and **(B)** corresponding results.

**FIGURE 4:**
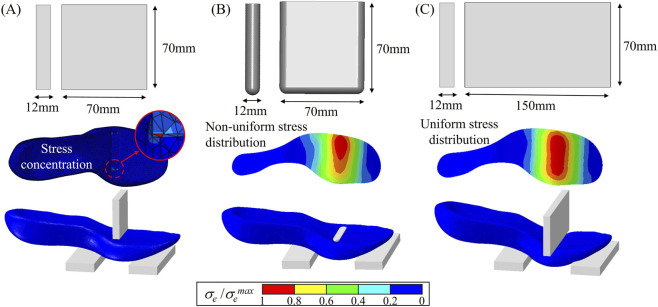
Evaluation of loading jig geometries in three-point bending simulation using FEA. **(A)** Conventional jig with sharp edges, showing stress concentration. **(B)** Rounded jig, resulting in non-uniform stress distribution. **(C)** Lengthened jig, achieving uniform stress distribution across the midsole.

In the bending model, the assembly consisted of the following components: midsole (carbon plate embedded in foam), loading jig, and two supports, as illustrated in Figure 3, where the loading jig and the two supports were modeled as rigid bodies. This simulation was designed based on the conventional LBS test utilizing a three-point bending setup, a methodology that has also been analyzed through FEA in a previous study (Guo et al., 2025). The distance between the two supports was determined according to established protocols in the literature, and the loading jig was positioned center of the forefoot (Roy and Stefanyshyn, 2006). The geometry of the loading jig and the magnitude of displacement were determined through preliminary simulations, as illustrated in Figure 4.

A conventional rectangular jig (12 mm thick and 70 mm wide), which has been widely employed in three-point bending tests for evaluating LBS (Roy and Stefanyshyn, 2006), presents two key limitations: a sharp edge induces local stress concentrations and produces a loading condition closer to indentation than true bending. Further, this design was not the result of a systematic design process. Instead, it originated from the initial proposal of the method and was adopted for decades without systematic validation. For this reason, FEA was systematically utilized in the present study to evaluate and refine the jig geometry. To address these issues, two alternative jig geometries were evaluated with FEA. First, a rounded jig was tested, which effectively reduced local stress concentrations. However, due to the asymmetric geometry of the midsole itself, this design resulted in non-uniform stress distribution across the carbon plate (Figure 4B). Subsequently, a lengthened jig (12 mm thick and 150 mm wide), covering the entire lateral width of the midsole was evaluated. This wider design ensured uniform stress distribution in the carbon plate and eliminated localized stress concentrations (Figure 4C). As a result, the lengthened jig was selected for subsequent simulations and validation experiments. Additionally, the edges of the two support fixtures were filleted with a radius of 0.5 mm to further prevent stress concentrations. After determining the geometry of the loading jig, the boundary conditions, particularly the magnitude of displacement of the loading jig, were decided. According to previous research, the displacement magnitude ranged from 7.5 mm to 15 mm, with 7.5 mm being the most commonly employed value (Ortega et al., 2021).

**FIGURE 5:**
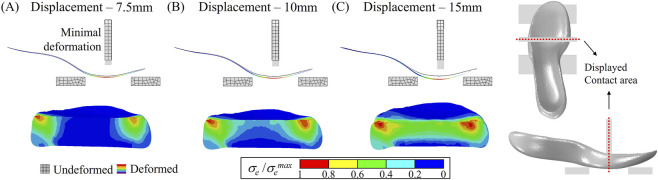
Evaluation of jig displacement magnitudes in three-point bending simulation using FEA. Simulated deformation of the carbon plate and transverse midsole cross-section under **(A)** 7.5 mm, **(B)** 10 mm, and **(C)** 15 mm of vertical displacement.

However, simulation results indicated that a displacement of 7.5 mm led to deficient deformation of the midsole, including the carbon plate, as illustrated in Figure 5A. Specifically, minimal deformation was observed in the carbon plate, with localized deformation occurring primarily in the midsole foam at lateral edges. A similar deformation pattern was observed under 10 mm displacement, as illustrated in Figure 5B. In contrast, a 15 mm displacement produced a uniform stress distribution throughout the midsole foam while avoiding excessive compression, indicating it as a more appropriate loading condition. Based on these considerations, a displacement magnitude of 15 mm was selected (Figure 5C) for the evaluation of LBS. In consequence, the loading jig with dimensions of 12 mm thick and 150 mm wide was displaced −15 mm in the *z*-direction, while the remaining 5 degrees of freedom (DOF) were fully constrained. The overall boundary conditions of the bending model, along with an example of resulting deformation, are illustrated in Figure 3A.

#### 2.3.3 Lateral loading model - rearfoot stability

**FIGURE 6:**
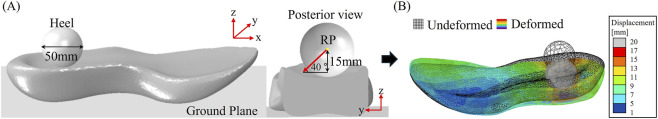
**(A)** Boundary conditions of the lateral loading model and **(B)** corresponding results.

In addition to energy return, stability is a critical factor for designing running shoes. The conventional method for evaluating the stability of shoes involves assessing torsional stiffness. However, as the torsional stress is primarily concentrated at the midpoint of the shoe, it does not directly correlate with the risk of ankle sprain (Zifchock et al., 2017). To address this limitation, a novel simulation approach that imitates an ankle sprain was employed, with a particular focus on assessing rearfoot stability. In the lateral loading model, which is detailed in Figure 6, the assembly consisted of the following components: midsole (carbon plate embedded in foam), heel, and ground plane, while the ground plane and heel were modeled as rigid bodies. The heel with a diameter of 50 mm was positioned in the rearfoot region to simulate the heel strike. The tip diameter was determined based on the measured width of the insole area corresponding to the heel contact region, provided in Supplementary Material A. Furthermore, to mimic the biomechanical impact of excessive supination, which is a major cause of ankle sprains during running, the indenting tip was displaced at 40° along the *z-*axis. This value intentionally exceeds the commonly reported ankle sprain threshold angles of about 30°, which is linked to both ankle sprains and normal ankle range of motion (Wright et al., 2000; Bok, Lee, and Lee, 2013), to represent an extreme loading scenario causing injuries. The indenting tip was displaced by −15 mm in *z-*direction, corresponding to approximately one-third of the thickness of the midsole, where the magnitude of the displacement was determined based on the strain level resulting from peak vertical ground reaction force during running from FEA simulation, as summarized in Supplementary Material B (Kluitenberg et al., 2012; Ito et al., 2017).

#### 2.3.4 Mesh sensitivity test

**FIGURE 7:**
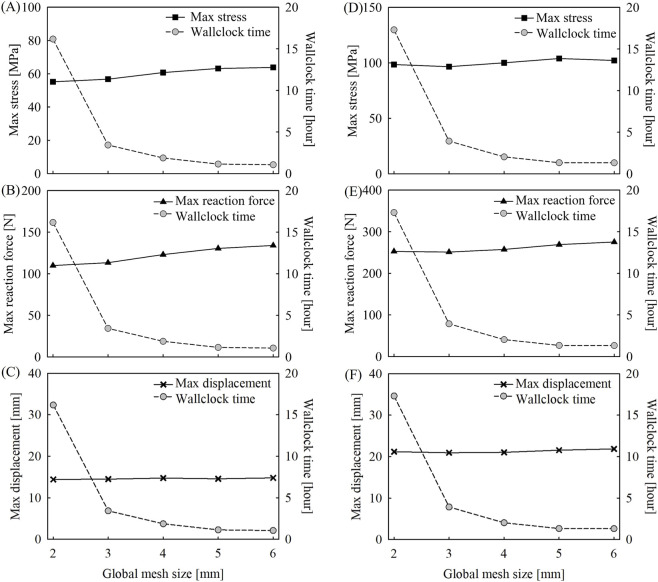
Effects of global mesh size on **(A,B)** maximum Von Mises stress, **(C,D)** maximum reaction force, **(E,F)** maximum displacement at midsole and computational time in bending and lateral loading model.

Prior to the simulations, a mesh sensitivity test was conducted for both bending and lateral loading models. The global mesh size ranged from 2 mm to 6 mm in increments of 1 mm. For all simulations, the identical global mesh size was applied to both the midsole foam and carbon plate. The results of the mesh sensitivity analysis are presented in Figure 7. A global mesh size of 4 mm was selected for both models, as it resulted in differences with the finest mesh model (2 mm) under 5% in both maximum stress and maximum reaction force. Additionally, the computation time approximately doubled when the mesh size changed from 4 mm to 3 mm, increasing from 6,735 s to 12,336 s for the bending model, and from 7,352 s to 13,861 s for the lateral loading model.

### 2.4 Experiments

**FIGURE 8:**
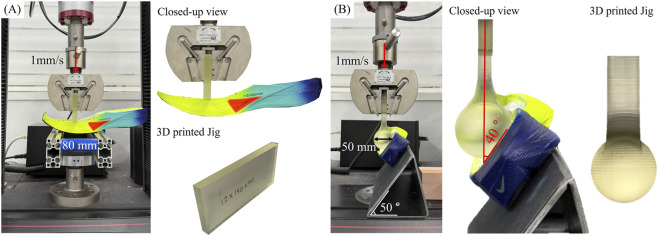
Experimental setup of **(A)** bending and **(B)** lateral loading models.

The two FEA models proposed in this study were validated with a universal testing machine (UTM) (Instron 5,969, Instron, Norwood, MA, United States), equipped with a 50 kN load cell, as illustrated in Figure 8. For experimental validation, only the midsole and outsole were tested, with the upper part of the shoe removed. This approach was taken to ensure clear and consistent contact with the midsole. Although the force intervention from the upper is minimal, it could still influence the result, even if the pre-load is applied. Furthermore, in real running situations, the foot is positioned between the midsole and the upper, making direct contact with the midsole. Thus, excluding the upper part reflects the load transfer more accurately relevant to actual use. To ensure proper fixation and contact of the midsole during testing, a preload of 5 N was applied in both experimental setups. The loading speed for both tests was set to 1 mm/s to minimize viscoelastic effects of the carbon plate and midsole foam, as only elastic and hyperelastic material properties were assigned in the FEA models. Each simulation was repeated for 20 cycles, and results were averaged for analysis. The jigs were 3D fabricated with the low force stereolithography (LF-SLA) Form3+ printer (Formlabs, MA, United States) with V4 Formlabs Clear resin (Formlabs, MA, United States) and washed for 15 min in an ultrasonic bath with isopropyl alcohol (99.9% concentration) twice, followed by post-curing at 60 °C for 80 min (Perin et al., 2023).

For validation of the bending model (Figure 8A), two aluminum supports were positioned at a fixed distance of 80 mm, consistent with the setup used in the FEA model. To minimize slippage during testing, sandpaper was attached between the supports and the UTM. For validation of the lateral loading model (Figure 8B), a 50-degree aluminum slope was fixed with UTM with screws, which can result in 40-degree displacement on midsole, consistent with FEA simulation and sandpaper was also attached to the inclined surface of the slope, ensuring stable contact with the midsole.

### 2.5 Design of experiments (DoE)

The three midsole foam materials of Table 2 (EVA, TPU, PEBA) have been combined with five carbon plate thicknesses (0.75, 1, 1.5, 2.25 and 3 mm) for a total of 15 cases for the bending and lateral loading models. The selection of thickness levels was based on the original carbon plate thickness of the NVF3, measured at 1.5 mm, which was subsequently multiplied and divided by factors of 2 and 1.5. For better readability of the graph, the results of 1.0 mm and 2.25 mm thickness levels are only presented in tables. The reaction force and internal energy of each FEA simulation are presented in the following chapter, allowing the identification of the combined effect of the midsole foam material and carbon plate thickness on both LBS and rearfoot stability. In turn, this analysis facilitated the identification of the optimal combination which allows sufficient energy return without compromising the runner’s stability.

## 3 Results

### 3.1 Validation

**FIGURE 9:**
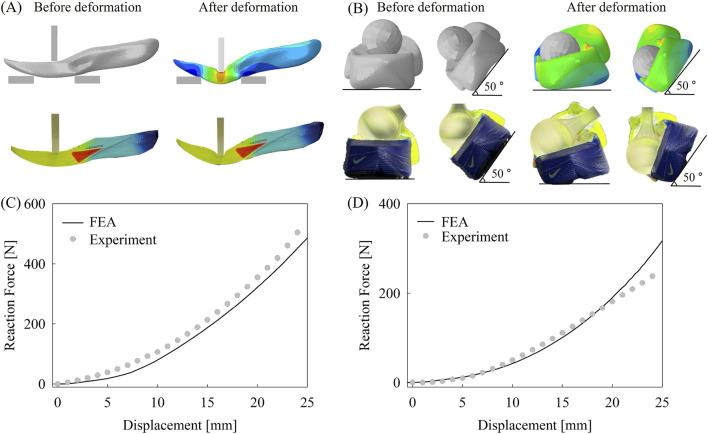
Validation of FEA models through comparison with experimental results. Comparison of geometry for **(A)** bending and **(B)** lateral loading model. Comparison of reaction force for **(C)** bending and **(D)** lateral loading model.

This section presents the validation results of bending and lateral loading FEA models. Validation was conducted by comparing FEA simulations with experimental data obtained using a UTM, as described in Sections 2.3 and 2.5, respectively. For both bending and lateral loading FEA models, a midsole with PEBA and a 1.5 mm carbon plate was adopted, which is identical to NVF3. The comparison of the deformed geometries of the midsole before and after deformation obtained from FEA and experiments is illustrated in Figures 9A,B. In addition, the comparison of reaction force from FEA (reference point of loading jig for bending model and heel for lateral loading model) and reaction force recorded by load cell in the experiment is illustrated in Figures 9C,D. The deviation of reaction force-displacement curve from UTM of repeated 20 cycles were under 2% of the corresponding average values in most ranges. To evaluate the robustness of the proposed FEA models, validations were performed under loading conditions that exceed the boundary conditions of the FEA models, confirming the generality of the model and showing that the model can work in a wider deformation range.

As illustrated in Figure 9, the proposed FEA models demonstrated good agreement with experimental results, with percentage errors ranging from 10% to 15% across most deformation ranges. The observed error may be attributed to the presence of an outsole and bonding layer in the physical specimen, which were not included in the FEA models, potentially leading to slightly higher reaction forces in the experimental results compared to the FEA results. However, shear simulation at displacements exceeding 20 mm exhibited larger errors, likely due to the use of midsole foam material properties derived exclusively from uniaxial compression tests. Since the lateral loading model involves multiaxial deformation, unlike the bending simulation, the lack of multiaxial material properties may have led to larger errors under large shear deformations. The geometry before and after deformation closely matched between FEA and experiment in both bending and lateral loading cases. Furthermore, the reaction force-displacement curve from FEA and experiment exhibited highly consistent behavior across a wide range of displacements, supporting the validity of the proposed models.

### 3.2 Longitudinal bending stiffness

**FIGURE 10:**
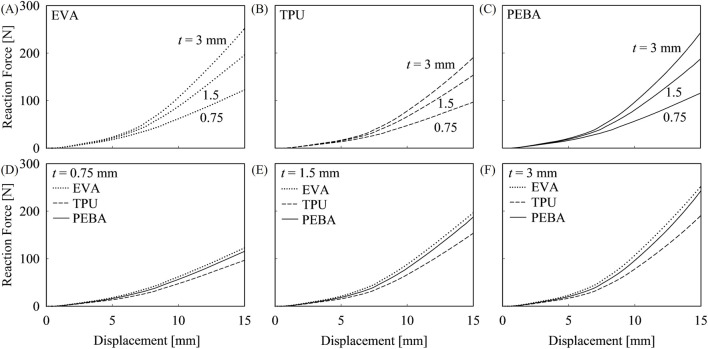
Bending reaction force with different midsole foam materials and carbon plate thicknesses. Graphs **(A–C)** show the reaction force for EVA, TPU, and PEBA, with different carbon plate thicknesses. Graphs **(D–F)** compare reaction forces of 0.75 mm, 1.5 mm, and 3 mm carbon plates across different midsole foam materials.

This section examines the bending reaction force across three different midsole foam materials and three carbon plate thicknesses. As illustrated in Figure 10, the bending reaction force increases with a thicker carbon plate across all midsole foam materials, indicating higher LBS of midsole. However, the increase in reaction force was less than proportional to the increase in thickness. Specifically, when the carbon plate thickness was doubled, the reaction force increased by approximately 60% and 30% for the thickness increased from 0.75 mm to 1.5 mm and from 1.5 mm to 3 mm, respectively. This indicates that excessively thick carbon plates may not be necessary to enhance LBS effectively. Notably, TPU exhibited the lowest sensitivity to changes in carbon plate thickness compared to EVA and PEBA. At a given carbon plate thickness, EVA demonstrated the highest bending reaction force across all thicknesses, which is comparable to PEBA, showing less than 10% differences under all conditions. In contrast, TPU exhibited the lowest reaction force among the three midsole foam materials, with differences ranging from approximately 20%–40% compared to the other two materials. These findings suggest that not only the carbon plate thickness but also the midsole foam material in carbon-plated running shoes plays a pivotal role in determining LBS. Moreover, the results suggest that adjusting the geometry of the carbon plate is not the only determining factor of adjusting target LBS, as the choice of midsole foam material also plays a significant role. Considering the results summarized in Table 3, the internal energy of carbon plate - midsole foam material combinations showed a consistent trend with reaction force.

**TABLE 3:** Summary of bending simulations with different foam-carbon plate combinations.

Midsole Foam material	Carbon plate thickness [mm]	Displacement 5 mm		Displacement 10 mm		Displacement 15 mm	
		Reaction force [N]	Internal energy [mJ]	Reaction force [N]	Internal energy [mJ]	Reaction force [N]	Internal energy [mJ]
EVA	0.75	18.73	26.08	62.62	191.40	122.76	592.23
EVA	1	20.08	28.37	72.72	218.36	150.11	715.04
EVA	1.5	21.92	31.53	88.96	257.65	196.48	911.58
EVA	2.25	23.28	33.91	102.10	286.52	234.58	1,064.29
EVA	3	24.14	35.08	108.28	298.96	251.92	1,128.72
TPU	0.75	14.06	19.07	47.99	139.04	96.64	447.44
TPU	1	14.89	20.60	55.74	158.93	119.06	546.42
TPU	1.5	16.02	22.54	67.05	184.73	153.50	686.63
TPU	2.25	16.98	23.98	75.38	202.29	179.37	784.61
TPU	3	17.41	24.66	79.11	209.87	190.68	824.35
PEBA	0.75	16.66	22.55	57.05	165.42	115.54	528.86
PEBA	1	17.71	24.40	66.41	188.60	141.97	639.13
PEBA	1.5	19.15	26.86	80.83	219.58	187.47	813.06
PEBA	2.25	20.36	28.71	92.22	242.81	225.20	944.58
PEBA	3	20.99	29.62	97.44	252.92	242.63	1,000.22

As carbon plate thickness consistently increases from 0.75 mm to 3 mm, internal energy at displacement of 15 mm rises about twice across all midsole foam material (EVA 191%, TPU 184%, PEBA 189%). This indicates that the TPU midsole exhibits the lowest sensitivity to changes in carbon plate thickness, implying that EVA and PEBA benefit more from carbon plate reinforcement. In terms of midsole foam materials’ comparison for the same carbon plate thickness, EVA consistently exhibited the highest internal energy, reaching 911.58 mJ at 15 mm of displacement with 1.5 mm carbon plate, which is 24.68% greater than TPU (686.63 mJ) and 10.81% higher than PEBA (813.06 mJ), highlighting its superior energy absorption capacity. The comprehensive graph of bending internal energy across different midsole foam materials and carbon plate thickness is provided in Supplementary Material C. Finally, higher reaction forces correlate with increased internal energy, as seen in EVA (196.48 N, 911.58 mJ), TPU (153.50 N, 686.63 mJ), and PEBA (187.47 N, 813.06 mJ), reinforcing that stiffer configurations store more energy, while softer materials dissipate it more efficiently. Furthermore, the results presented in Table 3 support the rationale for selecting an appropriate magnitude of vertical displacement in three-point bending simulations. At a displacement of 5 mm, the difference in reaction force and internal energy between carbon plates of varying thickness (a twofold increase) approximately ranged from 10% to 20%. When the displacement increased to 10 mm, these differences expanded to 15%–40%, and at 15 mm, the range further increased to approximately 25%–60%. This finding supports that 7.5 mm of vertical displacement may be insufficient to capture the influence of the carbon plate, thereby supporting the use of larger displacements for more in-depth analysis.

### 3.3 Rearfoot stability

**FIGURE 11:**
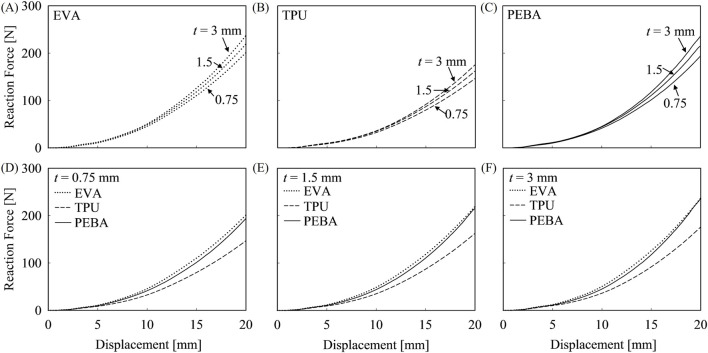
Reaction force in lateral loading model with different midsole foam materials and carbon plate thicknesses. Graphs **(A–C)** show the reaction force for EVA, TPU, and PEBA, with different carbon plate thicknesses. Graphs **(D–F)** compare reaction forces of 0.75 mm, 1.5 mm, and 3 mm carbon plates across different midsole foam materials.

In contrast to the bending simulation, the lateral loading model revealed no significant variation in reaction force among midsoles with varying carbon plate thicknesses within the same midsole foam material, as illustrated in Figure 11. This indicates that the thickness of carbon plates does not have a notable impact on rearfoot stability in lateral loading. Instead, the results suggest that the stability of carbon-plated running shoes is primarily determined by the midsole foam material rather than the thickness of carbon plates. Among the midsole foam materials, EVA and PEBA exhibited higher reaction force across all thicknesses, with about 25%–40% higher reaction forces compared to TPU midsoles. This indicates better stability of the EVA and PEBA midsole in lateral loading, which is particularly relevant for ankle sprain prevention. These findings suggest that EVA and PEBA are suitable midsole foam materials for enhancing the stability of carbon-plated running shoes.

Under lateral loading conditions, as carbon plate thickness increases from 0.75 mm to 3 mm, internal energy at max condition increases for all midsole foam materials, but with much smaller percentage changes compared to bending loading, ranging around 10%, as summarized in Table 4. This indicates that shear energy absorption is insensitive to variations in plate thickness. In addition to that, EVA maintains the highest internal energy in all thicknesses of carbon plate, reinforcing its superior energy absorption in shear loading as well. For ankle sprain prevention under lateral loading conditions, EVA is the best material for midsole, as it offers the highest resistance to shear forces and dissipates the most energy, effectively stabilizing the foot. Conversely, TPU is the weakest, as it provides the lowest resistance and energy absorption, making it more prone to lateral instability and ankle rolling.

**TABLE 4:** Results of lateral loading model with different foam-carbon plate combinations.

Midsole foam material	Carbon plate thickness [mm]	Displacement 5 mm		Displacement 10 mm		Displacement 15 mm	
		Reaction force [N]	Internal energy [mJ]	Reaction force [N]	Internal energy [mJ]	Reaction force [N]	Internal energy [mJ]
EVA	0.75	12.58	12.23	45.33	119.59	101.75	417.12
EVA	1	13.03	12.63	46.71	123.40	104.73	428.72
EVA	1.5	13.58	13.04	48.60	128.21	108.77	442.55
EVA	2.25	13.89	13.11	49.79	130.51	113.49	458.73
EVA	3	13.99	13.12	50.08	130.49	115.35	462.17
TPU	0.75	9.70	9.67	32.39	84.65	73.43	296.57
TPU	1	10.15	10.07	33.44	87.39	75.89	305.43
TPU	1.5	10.64	10.39	35.07	91.51	79.87	320.59
TPU	2.25	10.97	10.59	35.75	92.10	82.55	326.81
TPU	3	11.11	10.69	36.01	91.90	83.85	328.79
PEBA	0.75	11.56	10.98	41.09	107.80	93.00	368.36
PEBA	1	11.95	11.04	42.59	111.11	96.68	381.49
PEBA	1.5	12.37	11.88	43.83	113.38	100.80	390.72
PEBA	2.25	12.64	11.92	44.49	113.07	104.72	397.41
PEBA	3	12.74	11.93	44.79	112.85	106.83	400.36

The present findings indicate that carbon plate thickness is not a critical factor for stability in lateral loading models. However, current results should not be interpreted as suggesting that carbon plate thickness is irrelevant to all running-related injuries. Rather, the influence of carbon plate thickness appears limited in the specific context of lateral loading when plate geometry and position are held constant. However, variations in the carbon plate’s position within the midsole or its curvature could potentially affect stability under lateral loading.

## 4 Discussion

The results of this study show that both midsole foam material and carbon plate thickness influence LBS, aligning with prior research (Hoogkamer et al., 2018; Malisoux et al., 2020; Silva et al., 2009; Chambon et al., 2014). It is well established that a thicker carbon plate generally results in higher LBS. However, this study reveals that the relationship between carbon plate thickness and LBS is not strictly proportional (lower than proportional), suggesting that an excessively thick carbon plate may not be necessary for optimal running shoe design. Additionally, no prior studies have reported that EVA foam yields higher LBS compared to other midsole foam materials such as TPU and PEBA. Furthermore, a novel aspect of this study is the FEA-based modeling of lateral loading models with running shoes, which has not been explored in prior research. The findings indicate that, while the carbon plate has a negligible influence on the stability of running shoes under lateral loading conditions, the midsole foam material plays a significant role. Specifically, EVA demonstrated superior stability, exhibiting approximately 40% higher shear reaction force compared to TPU. This outcome is attributed to the inherent compressive properties of each midsole foam material.

From a practical perspective, these findings suggest that for amateur or beginner runners, for whom stability is a critical factor in selecting running shoes and injury risk (e.g., ankle sprain) is higher, carbon-plated running shoes with EVA foam would be a suitable choice. While PEBA is often preferred for its lightweight properties and high energy return (Aimar et al., 2024; Van Alsenoy, Ryu, and Girard, 2019; Brückner et al., 2010), EVA midsole is more recommended for enhancing stability and LBS simultaneously. However, for more professional runners, whose primary goal is to achieve even marginal performance improvements, a PEBA midsole with an adequate thickness of carbon plate may be a more suitable option. While the present study demonstrates EVA’s superior stability, particularly for amateur runners, this finding should be considered alongside the widespread use of PEBA-based midsoles in racing footwear. The greater stability can be attributed to its higher density, which increases midsole stiffness and limits excessive deformation, thereby enhancing stability. However, this higher density also increases shoe mass, which can reduce running economy, as shown in previous studies (Frederick, 1984). PEBA combines low density with high resilience, resulting in weight savings and improved running economy, as reported in metabolic studies (Frederick, 1984; Hoogkamer et al., 2018). Consequently, the choice of midsole foam material often represents a trade-off between stability and running economy, with EVA favoring the former and PEBA the latter, depending on the performance objectives of the target user group. In addition to performance-related considerations, the selection of midsole foam materials is also influenced by manufacturing constraints. PEBA, which offers low density and high energy returns, has relatively high production costs, which can limit its use in mass-market products. However, EVA is comparably cost-effective but has higher density and it is prone to show reduced cushioning performance over prolonged use. These practical factors mean that manufacturers must balance material performance with cost and expected product lifespan, leading to different material selections depending on the targeted runners. Additionally, although TPU showed lower LBS and stability than other materials, it offers higher resilience, durability, and temperature stability than EVA, helping maintain cushioning and rebound over time. These advantages make TPU a popular choice in commercial running shoes where long-term comfort and performance are important (Hoogkamer et al., 2018).

Although various critical aspects related to the design optimization of carbon-plated running shoes have been brought forward in this study, some limitations are still present. First, the material properties used in the FEA were obtained from existing literature rather than *in situ* measurement. This fact is mostly caused by the complexity of obtaining tensile or compressive specimens from commercial shoes’ midsoles, a fact which might cause slight variation in the local behavior, though the overall trend remains mostly the same. Additionally, the hyperfoam material modeling was based solely on the compressive stress-strain data, even though the model fitting was stable for all strain ranges, including tensile deformation, and validated with experimental results. Although the primary deformation mode agrees with compressive behavior, since both bending and lateral loading simulations involve tensile deformation, incorporating tensile stress-strain data would enhance the accuracy and reliability of the results. To this end, future research could improve the accuracy and depth of the analysis by directly characterizing the midsole foam materials through instrumented indentation testing (Quagliato et al., 2025b), from which both tensile and compressive behaviors can be identified, thereby improving the reliability of the results but still employing the proposed FEA modeling framework.

Nevertheless, while this study conducted dynamic simulations, the boundary conditions were limited to displacement constraints. The present models did not fully represent the dynamic conditions encountered during actual running. As noted in previous study, multiple factors can influence joint mechanics and injury risk, emphasizing the importance of developing personalized FEA models in designing running shoes (Kluitenberg et al., 2012). However, accurately simulating such conditions requires highly detailed geometries and material properties for soft tissues, bones, ligaments, tendons, and cartilage, along with precise specifications of boundary conditions, where most of them are challenging to validate experimentally. Given these limitations, a simplified modeling approach was adopted to investigate general biomechanical trends rather than replicate all aspects of real-world running dynamics. Nevertheless, characterizing joint interactions under dynamic running conditions remains an important research objective, and future research should focus on developing a comprehensive simulation model that integrates personalized geometries, material properties and boundary conditions (Quagliato et al., 2025a; Negishi et al., 2020; Suzuki et al., 2017; Quagliato et al., 2025b).

Furthermore, this study proposes a novel standard for conducting three-point bending tests for evaluating the LBS of footwear. While various traditional methods exist for assessing LBS, the most commonly employed approach is the three-point bending test, with different loading rates, displacement ranges, and jigs geometries (Ortega et al., 2021). The present study demonstrates that a sufficiently wide jig, spanning the entire lateral width of the shoe, enables uniform stress distribution across the midsole. In traditional methods, where narrow jigs are used (Guo et al., 2025; Roy and Stefanyshyn, 2006), deformation is highly concentrated under the contact region, leading to localized indentation-like behavior. Although the foot does not cover the entire width of the shoe during running, the midsole deformation is governed by bending rather than local indentation. This may be attributed to the geometric and material continuity of the midsole, which distributes force over a broader region under dynamic loading conditions. For a better reflection of the running scenario, the current study suggests a lengthened jig configuration, which can lead to more accurate evaluation of LBS, thereby offering a more appropriate and physically representative test setup for carbon-plated running shoes.

Moreover, the displacement range of three-point bending ranged from 7.5 mm to 15 mm, with the setting of 7.5 mm being the most widely employed. Based on FEA studies, this study recommends a vertical displacement of 15 mm as a more effective setting for evaluating LBS. However, the influence of loading rate was not explored in this study, as the FEA models incorporated only elastic and hyperelastic material properties and did not incorporate viscoelastic material properties, resulting in rate-independent results. In contrast, real-world running conditions involve dynamic loading scenarios where loading and deformation rates play a crucial role. Therefore, future research should focus on the loading rate dependency of LBS by incorporating time-dependent material models, such as viscoelastic material properties (Negishi et al., 2020) in combination with unloading simulation of during dynamic running.

In conclusion, both midsole foam material and carbon plate thickness influence LBS of carbon-plated running shoes. While LBS increases with greater carbon plate thickness, the relationship is less than proportional, suggesting that excessively thick carbon plates are not necessarily beneficial. Among the midsole foam materials employed in this study, EVA exhibited the highest LBS, followed by PEBA, with TPU demonstrating the lowest LBS. In contrast, carbon plate thickness hardly affected the rear foot stability of carbon-plated running shoes. Notably, EVA midsoles demonstrated exceptional stability, making EVA the optimal material for simultaneously enhancing both LBS and stability. The modeling approach proposed in this study defines a framework for the systematic evaluation of the LBS and rear foot stability in running shoes and, for this reason, can be of interest to biomechanical engineers involved in designing and optimizing shoes’ design.

## Data Availability

The original contributions presented in the study are included in the article/Supplementary Material, further inquiries can be directed to the corresponding author.
