# Supplementary material for: Finite element analysis-based optimization of longitudinal bending stiffness and rearfoot stability in carbon-plated running shoes
Source: Front Bioeng Biotechnol. 2026 Mar 30;14:1797727. doi: 10.3389/fbioe.2026.1797727 (PMC13071388; doi:10.3389/fbioe.2026.1797727)
Supplement: Supplementary file 1 [file Presentation1.pdf]

Supplementary Material

1 Supplementary Material A

The photos of NVF3 US M10 size (280 mm) including cross-sectional view and detailed geometry measurement are illustrated in Figure A.

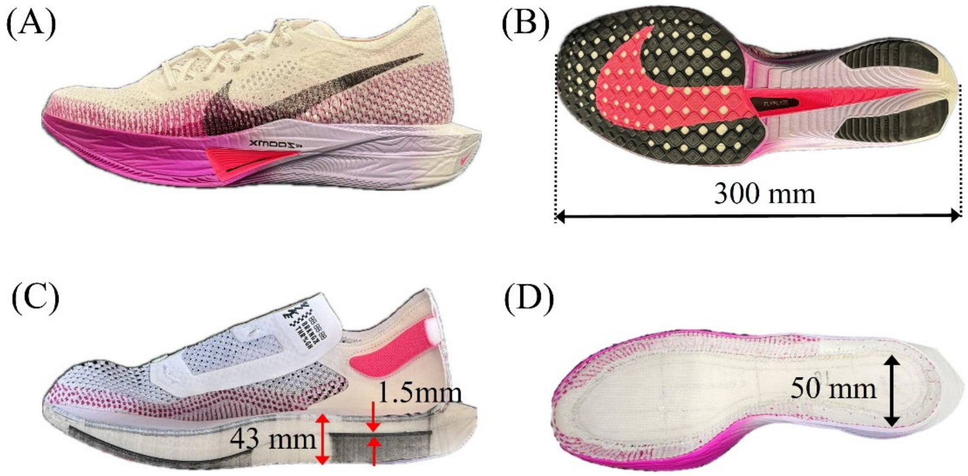

Figure A. (a) Side view, (b) Plantar view, (c) Cross-sectional view and (d) Top view of NVF3

2 Supplementary Material B

In the lateral loading model, the displacement magnitude in *z*-direction was set as -15mm. This value was determined based on a running scenario for an 80 kg male. According to previous research, the peak vertical ground reaction force (GRF) during heel strike can reach approximately 2 times body weight [1]. Accordingly, the estimated peak force applied to the shoe is approximately 1,500 N, calculated as 9.81 m/s^2 (gravitational acceleration) × 80 kg × 2. Based on this estimation, a boundary condition of 1,500 N vertical load was applied to the midfoot and rearfoot regions in the FEA simulation. As visualized in Figure B, the compressive strain across the midsole, ranged from 0.3 to 0.4. Based on this, the vertical displacement was set to 15 mm, corresponding to an estimated strain of 30-40% of the original thickness of midsole (43 mm), yielding a deformation range of 12.9 mm to 17.2 mm.

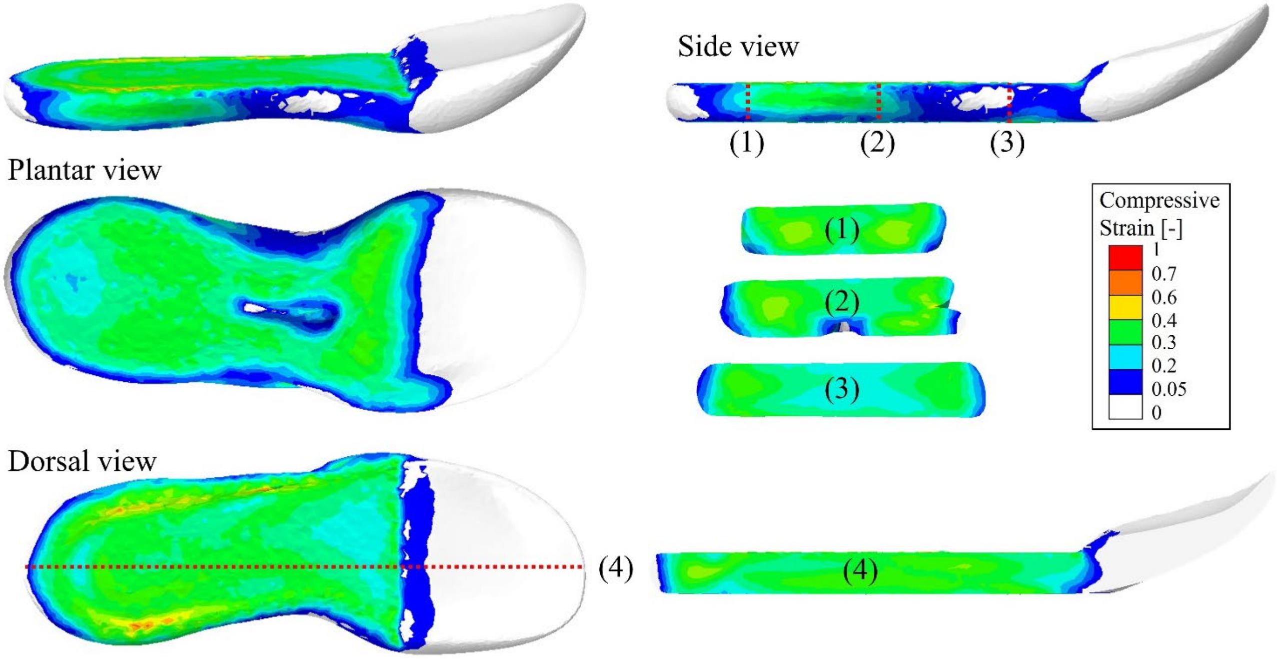

Figure B. FEA simulation of magnitude of compressive strain during running

[1] Kluitenberg, B., Bredeweg, S.W., Zijlstra, S. *et al.* Comparison of vertical ground reaction forces during overground and treadmill running. A validation study. *BMC Musculoskelet Disord* **13**, 235 (2012). https://doi.org/10.1186/1471-2474-13-235

3 Supplementary Material C

3.1 Figure C.1

Comprehensive graph of bending internal energy across different midsole foam materials and carbon plate thickness.

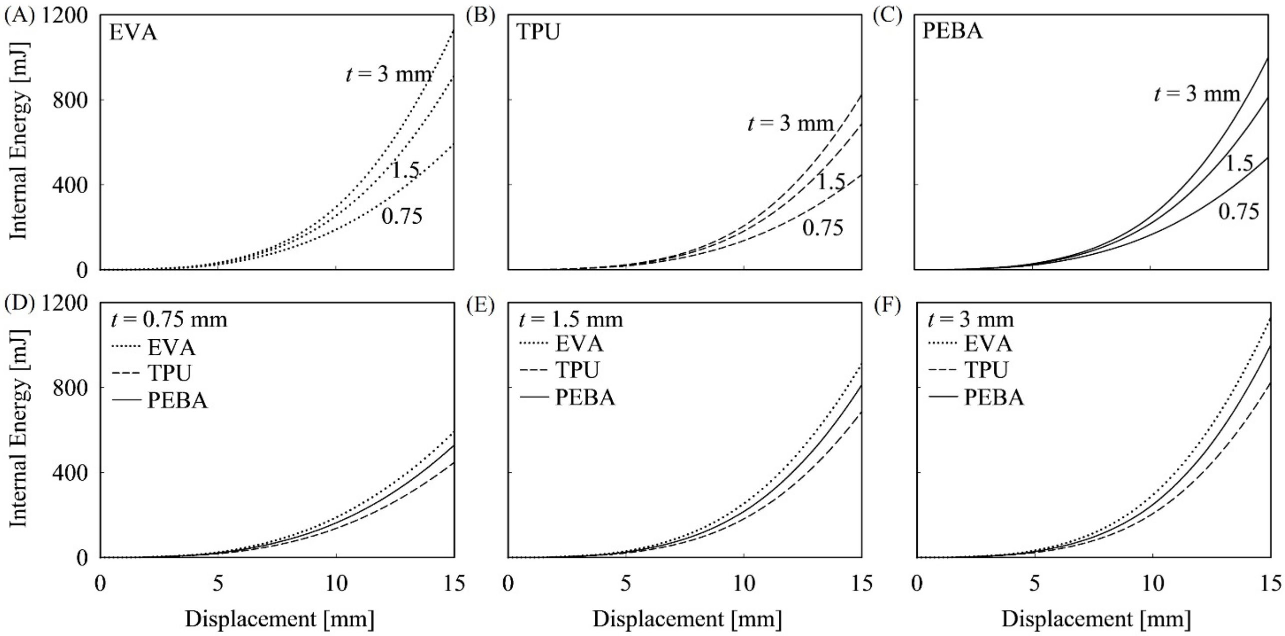

Figure C1. Bending internal energy with different midsole foam materials and carbon plate thicknesses. Graphs (a), (b), and (c) show the internal energy for EVA, TPU, and PEBA, with different carbon plate thicknesses. Graphs (d), (e), and (f) compare internal energy of 0.75 mm, 1.5 mm, and 3 mm carbon plates across different midsole foam materials

3.2 Figure C.2

Comprehensive graph of lateral loading internal energy across different midsole foam materials and carbon plate thickness.

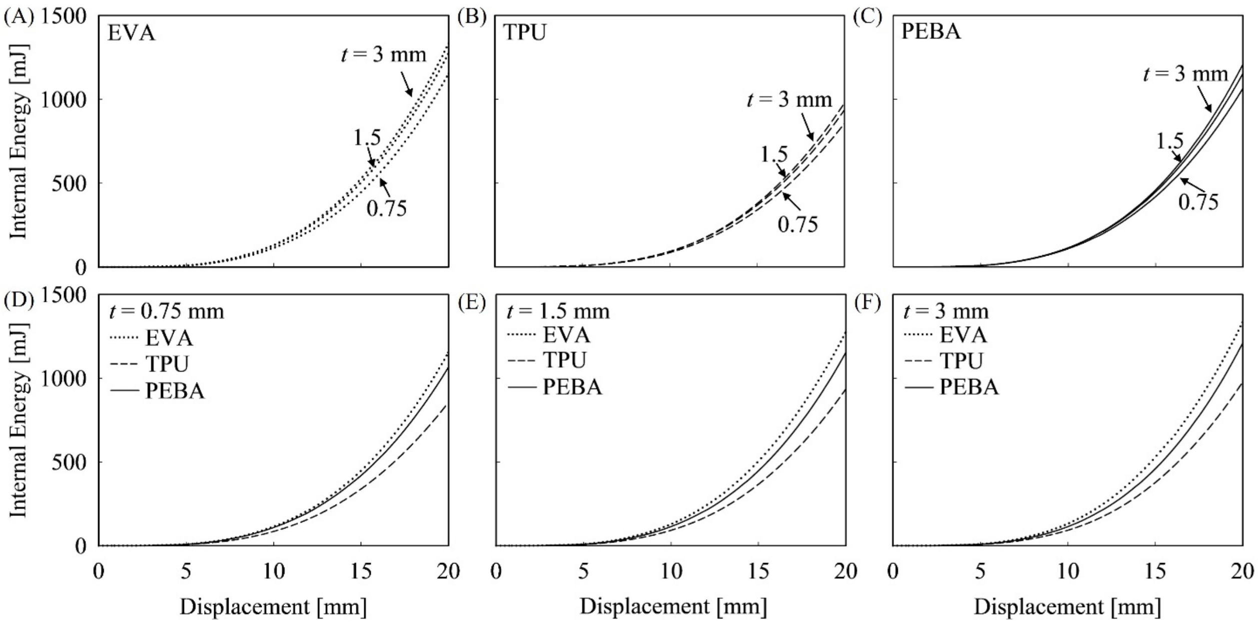

Figure C2. The ankle sprain internal energy with different midsole foam materials and carbon plate thicknesses. Graphs (a), (b), and (c) show the internal energy for EVA, TPU, and PEBA, with different carbon plate thicknesses. Graphs (d), (e), and (f) compare internal energy of 0.75 mm, 1.5 mm, and 3 mm carbon plates across different midsole foam materials
